# Serum Metabolomics of Senior Dogs Fed a Fresh, Human-Grade Food or an Extruded Kibble Diet

**DOI:** 10.3390/metabo15100676

**Published:** 2025-10-17

**Authors:** Ryan Yamka, Rae Sires, Joe Wakshlag, Heather J. Huson

**Affiliations:** 1 The Farmer’s Dog, New York, NY 10012, USA; rsires@thefarmersdog.com (R.S.); drjwakshlag@thefarmersdog.com (J.W.); 2 Department of Animal Science, Cornell University, Ithaca, NY 14853, USA; hjh3@cornell.edu

**Keywords:** senior dog, metabolomics, canine metabolomic profile, human grade fresh food, advanced glycation end product, AGE

## Abstract

**Background/Objectives**: Despite the growing popularity of fresh food for dogs, there is an extremely small amount of literature evaluating the potential health benefits of fresh food and reduced processing compared to traditionally processed shelf stable cans, extruded kibble, or other food formats. Additionally, aging dogs have been previously documented to have altered metabolism and nutritional needs compared to a healthy adult dog population, but these differences are not well defined. The objective of the study was to compare the effects of feeding a fresh, human-grade food versus a standard extruded kibble diet in a year-long longitudinal study on serum metabolomic profiles in senior dogs. **Methods**: Twenty-two healthy mixed-breed geriatric Alaskan sled dogs were age- and sex matched into two feeding groups. All dogs were fed the extruded diet (control) for a 4-month washout period prior to being transitioned into their respective treatment group. Group 1 continued to be fed the control diet, while Group 2 was transitioned to a fresh, human-grade food (treatment). Body weight and body condition were assessed monthly, and calorie intake was adjusted to maintain body weight. Individual serum samples were collected at day 0 and months 1, 3, 6, and 12. Metabolomic profiling of serum samples was performed by Metabolon, Inc. (Durham, NC, USA). Data was analyzed using two-way analysis of variance with repeated measures to determine treatment differences. **Results**: Dogs fed the treatment food had an increase in branched-chain amino acid metabolism, creatine, carnosine, anserine, fatty acid metabolism, long-chain n-3 fatty acids, lipolysis, and ketogenesis. The treatment group had decreased advanced glycation end products, fatty acid synthesis, and creatinine. **Conclusions**: This study is the first long-term feeding study evaluating serum metabolomics in dogs that demonstrates the dramatic and sustained impact that diet can have on canine metabolism.

## 1. Introduction

The pet food industry is an evolving landscape fueled by consumer demands and offers a wider range of specialty products and food formats than ever before. In human literature, there have been many large epidemiologic studies that demonstrate a potential link between consuming highly processed foods and the development of chronic diseases [1]. As humans consider the potential health implications of consuming highly processed food for themselves, pet owners also have similar concerns for their companion animals. The desire to provide complete and balanced dog and cat foods from whole food sources and with less processing and artificial preservatives is rapidly growing [2]. Despite the growing popularity of fresh food for dogs, there is an extremely small amount of literature evaluating the potential health benefits of fresh food and reduced processing compared to traditionally processed shelf stable cans, extruded kibble, or other food formats. Adding additional complexity to this lack of scientific evaluation, definitive nutritional requirements of aging or senior dogs have not been established [3,4].

Aging is a spectrum of physiologic changes and the reduced ability of the body to respond and repair from injury that ultimately result in altered tissue function. Over time, these additive biologic changes predispose an animal to the development of chronic disease [5]. Senior dogs, generally defined as animals that are 7+ years of age, have been previously documented to have altered nutritional needs relative to a healthy, younger adult dog, including reduced energy requirements and the potential for an increased total protein requirement [6,7]. Generally, macronutrient and mineral apparent digestibilities seem to be well maintained in aging dogs [8].

Metabolomics offers unique insights into an organism’s physiologic state and has been previously applied to healthy young, adult, and senior dog populations to help gain a better understanding of age-associated metabolic changes in senior dogs. Metabolic profiles have been previously evaluated, comparing healthy juvenile and adult dogs, and identified several metabolites that were significantly associated with age, which primarily involved amino acid metabolism and bile secretion pathways [9]. Glutamine was consistently higher, and N-acetylhistamine and uracil concentrations were lower in the adult group compared to younger animals [9]. One recent study by Jackson evaluated the impact of three diets with differing macronutrient profiles: one with high carbohydrate content as a baseline control diet and the two treatment groups having either a high protein or a high fat content with low carbohydrate content in a crossover study design [10]. The researchers concluded that the diet containing the high fat content resulted in dogs deriving energy from fatty acids and beta-hydroxybutyrate, and a reduction in carbohydrate content may potentially benefit dogs based on improvements to metabolism, and foods with this macronutrient distribution are safe and effective for healthy adult dogs. The study evaluated healthy older-adult beagle dogs (mean age and weight of 7.1 years and 10.3 kg, respectively), which may not be reflective of metabolic changes or adaptation that occurs in geriatric (>12 years of age) or larger-breed dogs [10]. Other previous studies that have evaluated senior dogs’ metabolomic profiles, either in home environments or in controlled laboratory settings, were not designed to effectively evaluate the impact of diet processing or macronutrient distribution on serum metabolomics [11,12,13].

Previous studies have evaluated potential metabolomic differences in dogs diagnosed with arthritis, sepsis, and canine cognitive dysfunction compared to healthy controls, which may allow for the development of new medical or nutritional interventions [14,15,16]. Untargeted metabolomics was used to evaluate the impact of supplementation of medium-chain triglycerides on systemic metabolism in a crossover design in medium- and large-breed young and senior dogs [17]. It demonstrated alterations in circulating lipids, ketone bodies, glutamine, and select amino acids, which may have potential health benefits [17].

Finally, consuming foods that are classified as ultra-processed has been previously documented to have a measurable impact on the metabolome in humans; however, the authors are unaware of any studies that have evaluated the impact of feeding a fresh, human-grade food compared to a highly processed, extruded kibble diet on serum metabolites in dogs [18]. Due to species differences in metabolism and nutritional requirements, studies evaluating the impact of ultra-processed food in dogs are warranted. Additionally, metabolomic data of larger-breed, senior dogs or geriatric dogs (>12 years of age) is limited. The objective of this study was to compare the effects of feeding a fresh, human-grade food versus an extruded kibble diet in a year-long longitudinal study on serum metabolomic profiles in senior, medium- to large-breed dogs in a controlled feeding environment.

## 2. Materials and Methods

All procedures were approved by the Cornell University College of Veterinary Medicine Institutional Animal Care and Use Committee (2018-0022) prior to study initiation. All methods were performed in accordance with the United States Public Health Service Policy on Humane Care and Use of Laboratory Animals.

Dogs and Diets: Twenty-two healthy mixed-breed geriatric (>12 years of age; 22.4 ± 3.2 kg; BCS 5/9) Alaskan sled dogs were age- and sex matched into two feeding groups at the Cornell University College of Veterinary Medicine. Prior to the start of the study, all dogs were fed a standard extruded, dry kibble diet (Lab Diet^®^ High Density Canine Diet 5L18, LabDiet, Richmond, IN, USA) for four months. Group 1 was continued on the standard extruded, dry kibble diet (control), while Group 2 was transitioned to a complete and balanced, fresh, human-grade food, The Farmer’s Dog Chicken Recipe (treatment). All dogs were weighed and given body condition scores monthly, and their daily calorie intake was adjusted as needed to maintain body weight and ideal body condition. Both diets met canine nutritional requirements for all life stages based on the Association of American Feed Control Officials published guidelines. Refer to Table 1 for control food and treatment food nutritional and ingredient information details.

Canine Inclusion and Exclusion Criteria: All dogs had a routine health assessment performed, including physical examination, complete blood counts (CBC; performed using the ADVIA 2120), and serum biochemistry profiles (Cobias C501) prior to being included in the study. Dogs were excluded from the study if blood work results showed evidence of kidney disease, liver failure, and/or endocrine disorders. Two dogs were included in the study that were previously diagnosed with an isolated, focal neoplasia (complete surgical excision; no evidence of recurrence over a 12-month period). Dogs with a history of systemic neoplasia were excluded from the study.

Feeding and Housing: All dogs were fed once daily throughout the duration of the study. Dogs were individually housed and let out in preformed groups of 5–8 dogs based on social dynamics (no aggressive or dominant behaviors towards specific dogs) into a 3-acre fenced area for 30 min twice daily to allow appropriate social interactions, self regulated exercise, and environmental enrichment. Dogs were allowed to interact and socialize with no encouragement to exercise, and most dogs would spend considerable time lying down outside in the grass. Occasional grass ingestion was noted but was not regularly observed. Dogs were allowed access to the outside between the temperatures of −5 °C and 30 °C. During days of inclement weather, dogs were allowed similar time to interact for enrichment in a 15 × 15 m room for up to 20 min. All dogs were kept up to date on routine vaccinations. Due to environmental exposure, all dogs received monthly flea, tick, and heartworm preventative medications throughout the duration of the study.

Metabolomic Sampling and Statistics: Individual dog serum samples were collected at day 0 and months 1, 3, 6, and 12. Samples were collected and stored at −80 °C until reaching the final time point. All samples were blinded and submitted together for third party metabolomic profiling (Metabolon, Inc., Durham, NC, USA).

Samples were prepared by Metabolon, Inc, using the automated MicroLab STAR^®^ system (Hamilton Company, Reno, NV, USA). Several recovery standards were added prior to the first step in the extraction process to ensure quality control. Proteins were precipitated with methanol via vigorous shaking for 2 min (GenoGrinder 2000, Glen Mills, Inc., Clifton, NJ, USA) followed by centrifugation. The resulting extract was divided into multiple fractions to allow for sample analysis and retention. Two fractions were analyzed by separate reverse phase ultrahigh performance liquid chromatography–tandem mass spectroscopy (RP/UPLC-MS/MS) methods with positive ion mode electrospray ionization (ESI), one for analysis by RP/UPLC-MS/MS with negative ion mode ESI, and one for analysis by hydrophobic interaction liquid chromatography (HILIC)/UPLC-MS/MS with negative ion mode ESI. The remaining fractions were reserved for backup. Samples were placed briefly on a TurboVap^®^ (Zymark, Hopkinton, MA, USA) to remove the organic solvent. The sample extracts were stored overnight under nitrogen before preparation analysis.

Quality control was performed using both internal laboratory-developed standards and study-specific endogenous metabolites using pooled serum samples. Several types of controls were analyzed in concert with the experimental samples: a pooled matrix sample generated by taking a small volume of each experimental sample (or, alternatively, use of a pool of well-characterized human plasma) served as a technical replicate throughout the data set; extracted water samples served as process blanks; and a cocktail of quality control standards that were carefully chosen not to interfere with the measurement of endogenous compounds were spiked into every analyzed sample, allowing instrument performance monitoring and aiding in chromatographic alignment.

Kyoto Encyclopedia of Genes and Genomes and Human Metabolome Database identifiers were used to cross-reference metabolites with known compounds. Data was analyzed using two-way analysis of variance (ANOVA) with repeated measures to determine whether the means of two populations were different. ANOVA was performed by Metabolon, Inc., using R packages (version 4.3.2) ImerTest and emmeans. The statistical model performed was Treatment2 + Time + Treatment2: Time + (1|Animal). Metabolites having a *p* < 0.05 (following a false discovery rate adjustment) were considered different among the two groups. Welch’s t-tests were conducted for inter-group comparisons (e.g., Test/Ctrl), and matched pairs t-tests for intra-group comparisons (e.g., Ctrl_D28/Ctrl_D0).

## 3. Results

### 3.1. Canine Population Health Overview

All dogs had physical examinations performed prior to the initiation of diet change and again at the end of the one-year dietary intervention. Systems assessed were nervous, musculoskeletal, dental, peripheral lymph nodes, abdominal organs, cardiovascular, thoracic auscultation, and integument. Many dogs exhibited lumbar or hip pain during musculoskeletal evaluation, and all dogs had evidence of periodontal disease, consistent with their age. Dermal cysts or lipomas were identified in some dogs, and none showed progression through the study duration that required medical or surgical intervention. The remainder of the organ system findings were within normal limits.

All dogs remained systemically healthy through the 1-year evaluation period and completed all diagnostic test sampling timepoints. Trended CBC and serum biochemistry profiles demonstrated mild fluctuations over time, consistent with the samples being from a senior population, but did not indicate the development of advanced systemic illness, which would have prompted removal from the study. Dogs were administered medications to manage acute or chronic conditions as determined appropriate by the attending veterinarian. Levothyroxine was administered to one dog for medical management of hypothyroidism. During the year-long period, one dog in the treatment group developed cholangiohepatitis (treated with enrofloxacin for 4 weeks), and two dogs (one in each group) showed forelimb musculoskeletal issues that resolved with short courses of carprofen and gabapentin. Monthly body condition score assessment variability was low, with dogs averaging a change of +/− 0.5 point on a 9-point scale over the duration of the study.

### 3.2. Metabolomic Serum Profile Results

Metabolomic profiling of the serum identified a total of 1196 metabolites (1036 named and 160 unnamed after cross-referencing with the two commercial databases). All identified metabolites, both named and unnamed, were included in the statistical analysis of the data to most thoroughly evaluate biochemical differences between the two treatment groups. Of these, 273 metabolites had a main treatment effect, 702 had a main time effect, and 305 had a combination treatment and time interaction.

### 3.3. Principal Component Analysis

**Figure 1:**
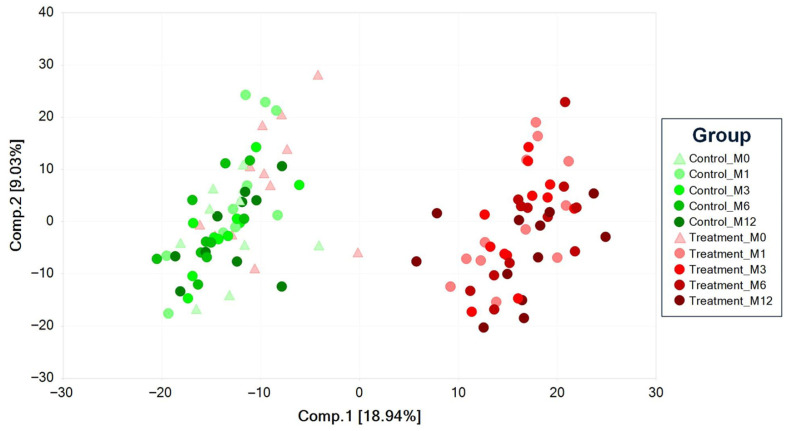
PCA of senior dogs fed a kibble diet (control) or fresh food (treatment).

Principal component analysis (PCA) is an unsupervised analysis that reduces the dimension of the data. Each principal component is a linear combination of every metabolite, and the principal components are uncorrelated. The number of principal components is equal to the number of observations. The first principal component is computed by determining the coefficients of the metabolites that maximize the variance of the linear combination. The second component finds the coefficients that maximize the variance with the condition that the second component is orthogonal to the first. The third component is orthogonal to the first two components, and so on. The total variance is defined as the sum of the variances of the predicted values of each component (the variance is the square of the standard deviation), and for each component, the proportion of the total variance is computed. For example, if the standard deviation of the predicted values of the first principal component is 0.4 and the total variance = 1, then 100 × 0.4 × 0.4/1 = 16% of the total variance is explained by the first component. Since this is an unsupervised method, the main components may be unrelated to the treatment groups, and the “separation” does not give an estimate of the true predictive ability. The percent variance explained is shown on the axes labels of the PCA plot in Figure 1 (PC1 = 18.94% and PC2 = 9.03%).

The PCA scores plot of all the samples is shown in Figure 1. The monthly serum samples from the dogs consuming the control diet (M1, M3, M6, and M12, respectively; indicated by green markers) are on the left side of the plot, while the corresponding samples from the dogs consuming the treatment diet (red markers) are on the right side of the plot. The dog serum samples from the treatment diet at time point 0 (M0; red triangle markers) are on the left side of the plot and overlap significantly with the control diet group at all time points, which is expected as all dogs had been fed the control diet for the 4-month washout period at the M0 time point. While there is no discernable trajectory in the samples from the control animals at any time point, there is a rightward trajectory over time in the M1, M3, M6, and M12 samples from the treatment diet animals. There was a large change in the serum metabolome within the first month of consuming the treatment diet, with changes in the metabolome over the rest of the year being modest in comparison to the first month.

### 3.4. Overview of Impacted Metabolic Pathways

Dogs fed the fresh, human-grade food had significant alterations in their amino acid metabolism compared to the control group, which was primarily evidenced by branched-chain amino acid catabolism (Table 2), fatty acid metabolism, lipolysis, ketogenesis (Table 3), and long-chain n-3 fatty acids (Table 4). Additionally, the treatment group had decreased creatinine, sugars, advanced glycation end products, metabolites commonly formed from consuming plant-based materials (Table 5), and significant changes to metabolites associated with oxidative stress (Table 6).

## 4. Discussion

### 4.1. Branched-Chain Amino Acid (BCAA) Metabolism

Differences in dietary nutritional profiles are expected to impact energetic pathways. The control diet and treatment diets varied in their macronutrient distributions, with the fresh, treatment food group providing more calories from proteins and fats and fewer from carbohydrates compared to the control group. This difference was expected to result in the treatment group utilizing fats and proteins, rather than carbohydrates, as sources of energy. One metabolic pathway in which amino acids can be utilized for energy generation is BCAA catabolism. The BCAAs leucine, isoleucine, and valine are important constituents of proteins and can serve as a crucial source of fuel that can be utilized to meet energy demands. BCAAs are first degraded by branched-chain aminotransferase (BCAT) to their α-keto acid derivatives and are further metabolized by the branched-chain α-keto acid dehydrogenase (BCKD) complex to produce metabolites that can enter anabolic pathways (i.e., gluconeogenesis or fatty acid synthesis) or pathways of energy generation (i.e., the tricarboxylic acid (TCA) cycle). A buildup of α-keto acid derivatives, whether due to high levels of BCAA catabolism or low BCKD activity, can lead to further metabolism of the α-keto acids to α-hydroxy acids. The BCAAs, their α-keto acid derivatives, and their α-hydroxy acid derivatives were generally higher in the samples from the treatment diet dogs compared to the control group, suggestive of higher BCAA availability and BCAA catabolism. An increase in BCAA catabolism, as seen in the dogs fed the treatment food compared to the control, may be suggestive of higher endogenous protein and amino acid catabolism in those dogs, or it may merely be reflective of a diet higher in protein or protein bioavailability in general, or BCAAs specifically. Both diets had similar total concentrations of BCAAs on a calorie basis (treatment—16.1 g per 1000 kcal; control—15.0 g per 1000 kcal). Considering the lack of exercise, which induces insulin sensitivity, and lack of global serum amino acid changes, the increase in BCAA catabolism is suggestive of dietary influences rather than muscle metabolic/catabolic differences [19]. This supports a difference in diet digestibility and amino acid bioavailability between the two diets driving the changes seen in BCAAs between the groups rather than a difference in total dietary concentrations of BCAAs.

One function of the urea cycle is the elimination of ammonia derived from amino acid catabolism. Urea was higher in samples from dogs fed the treatment diet compared to the control diet (not all time points being statistically significant), suggestive of some level of higher amino acid catabolism in the treatment diet dogs to generate glucose and citric acid cycle precursors. A urinalysis to support alterations in BCAA metabolite urinary excretion was not performed in this study. In addition, dogs on the fresh food diet had a higher overall creatine from baseline, which was likely from the creatine in lightly cooked food, as highly processed foods often produce creatinine, which cannot be utilized by skeletal muscle tissue [20]. Dietary creatine concentrations were not measured in this particular study.

### 4.2. Fatty Acid β-Oxidation and Fatty Acid Synthesis

Differences in the diet and intestinal microbiome could impact dietary fat digestion and uptake, which could result in an alteration in fatty acid metabolism. Fatty acids can be cleaved from triacylglycerols (TAG, fats), diacylglycerols (DAG), and monoacylglycerols (MAG), yielding DAG, MAG, and glycerol, respectively, in addition to the cleaved fatty acid. The glycerol backbone can enter the glycolytic pathway via glycerol 3-phosphate, which, through feeding a portion of the glycolytic pathway, can serve as a marker of lipolysis. Compared to the control diet group, the dogs fed the treatment diet had higher glycerol and glycerol-3-phosphate, which may be indicative of increased lipolysis.

Fatty acids are metabolized via β-oxidation to yield acetyl-CoA, which can feed into the TCA cycle. Individual fatty acids can be synthesized and elongated to support the synthesis of DAGs, necessary for the synthesis of TAGs for energy storage and phospholipids for membrane synthesis. During β-oxidation, fatty acids are conjugated to carnitine to facilitate their transport into the mitochondria. Thus, changes in acylcarnitine levels can be indicative of alterations in β-oxidation. In the samples from the treatment diet dogs, both fatty acids and acylcarnitines were generally increased compared to the control group. The short-chain fatty acid (SCFA) butyrate/isobutyrate was also higher in the samples from the treatment diet dogs. Many plant-derived fibers are digested by intestinal microbial populations to produce SCFAs, which can then be taken up by digestive tissues to provide energy for the host. SCFAs are also a primary energy source for colonocytes, and they have been shown to provide anti-inflammatory and immunological effects [21,22]. The increased level of this particular SCFA may be indicative either of an enhanced gut microbiome or may be reflective of a difference in fatty acid β-oxidation. Future evaluation of SCFA concentrations or the gastrointestinal microbiome may help to elucidate this mechanism.

3-Hydroxybutyrate (BHBA), a ketone body and end-product of fatty acid β-oxidation, was increased in the samples from the treatment diet dogs compared to the control group. When dogs are fed a reduced carbohydrate and increased fat food, the body produces BHBA as a result of metabolically transitioning from glucose to fat and fatty acids for energy. The increase in BHBA was an expected finding due to the treatment group having a lower carbohydrate content compared to the control group (3.8 vs. 110.3 g per 1000 kcal, respectively). Additionally, the fat content in the treatment group was higher than in the control (66.4 vs. 53.2 g per 1000 kcal, respectively). Jackson (2022) studied the effects of feeding foods to dogs with differing macronutrient distributions, similar to the foods evaluated in this study [10]. The high-carbohydrate food had a protein–fat–carbohydrate ratio of 25:37:38, while the low-carbohydrate and high-fat food had a ratio of 27:69:5 [10]. Similarly to our findings, Jackson (2022) saw an increase in BHBA, fatty acids, and acylcarnitines in the dogs fed the low-carbohydrate, high-fat food [10]. However, they did not see this occur in dogs fed the low-carbohydrate, high-protein food (macronutrient ratio of 53:39:8) [10]. Lastly, that study also found that feeding a low-carbohydrate, high-fat food was safe and effective in dogs with changes in metabolism that may be beneficial for dogs requiring a ketogenic food for diseases like cancer and diabetes. Tavener et al. (2024) studied the immune-modulating effects of feeding a low-carbohydrate and high-fat food (ratio of 27:69:5) on whole blood samples when compared to a high-carbohydrate food (25:37:38) in dogs [23]. Similarly to our findings, Taverner et al. saw increases in BHBA in the dogs fed the low-carbohydrate, high-fat food [23]. In addition, those authors observed a reduction in primarily inflammatory genes, indicating that the low-carbohydrate food provided anti-inflammatory benefits, which they concluded could be beneficial as a nutritional strategy for inflammation in dogs for obesity, dermatitis, renal disease, inflammatory bowel disease, and cancer [23].

Taken together, the elevations in BHBA, acylcarnitines, glycerol, and glycerol 3-phosphate are suggestive of increased lipolysis and fatty acid β-oxidation in dogs fed the treatment diet. Lastly, the treatment group had decreased levels of malonate when compared to the control-fed dogs, which is indicative of fatty acid synthesis occurring in the higher-carbohydrate-consuming kibble group.

### 4.3. Long-Chain Fatty Acid Metabolism

Omega-3 polyunsaturated fatty acids (PUFAs) are generally considered to be beneficial lipids, as they are typically associated with the production of anti-inflammatory molecules. Mammals are unable to synthesize omega-3 (and omega-6) PUFAs and must obtain them through their diet. Fish are a particularly abundant source of eicosapentaenoate (EPA) and docosahexaenoate (DHA) since the diet of many fish consists of algae that are rich in EPA and DHA. Some PUFAs, such as the omega-3 PUFAs EPA, docosapentaenoate (DPA), and DHA, were higher in the samples from the treatment diet animals compared to the control (not all statistically significant). Although fish oil was included in the treatment food and not the control food formulation, it is not likely to be the only reason for the increased levels of omega-3 fatty acids. The treatment food also contained chia seeds, which are an excellent source of alpha-linolenic acid (ALA). Previous studies in dogs have found that feeding ingredients rich in ALA, such as flax, has resulted in increased levels of ALA, EPA, and DPA but not DHA in the blood [24,25,26]. It has been shown that ALA alone can result in reduced expression of the inflammatory genes similar to EPA and DHA in dogs [26]. Additionally, ALA has been shown to improve transepidermal water loss in the skin and is beneficial for skin barrier function and potentially pruritus [24]. Both EPA and DHA have been shown to be beneficial for inflammation, osteoarthritis, and cognitive function [27,28,29,30].

### 4.4. Sugars and Advanced Glycation End Products (AGEs)

When comparing the samples from the dogs consuming the treatment diet to samples from the control diet animals, sucrose, a glucose-fructose dimer commonly found in plants, was lower in the treatment diet animals, while mannose was higher. Mannose is important for protein glycosylation, and digestion of food high in glycoproteins may increase systemic concentrations of mannose.

1,5-anhydroglucitol (1,5-AG) is a poorly metabolized dietary monosaccharide and a biomarker for short-term glycemic control. 1,5-AG was lower in the dogs fed the treatment diet, which may have been the result of more than one mechanism. The lower concentration of this metabolite may simply be indicative of the dietary 1,5-AG difference between the two diet groups and the treatment food having a low total carbohydrate content. Alternatively, it may have been lower due to competitive inhibition in the kidney. 1,5-AG is filtered by the renal glomerulus, and the majority is reabsorbed in the proximal tubule and returned to systemic circulation. Glucose is a competitive inhibitor of this reabsorption pathway. Due to this interaction, lower 1,5-AG blood concentrations may be indicative of poor glycemic control, and sustained increased concentrations of blood glucose increase urinary excretion of 1,5-AG.

The manufacturing process of ultra-processed pet foods (i.e., kibble and retorted) undergoes a high-heat thermal processing, which is used as a kill step to improve shelf life and alter the texture of the foods [31,32]. Temperature and residence time during food processing are known to influence Maillard reactions (MR), in which a reducing sugar binds to a free reactive amino group of an amino acid [31]. Lysine is one amino acid that may be impacted by the MR. As a result, lysine is irreversibly bound to dietary sugars and no longer available for metabolism due to the formation of AGEs, including pyrraline and Nɛ-(carboxymethyl)-lysine (CML) [31,32]. Dogs consuming the minimally processed treatment food had lower levels of both pyrraline and CML when compared to the control diet group. AGEs are currently being studied in dogs and humans and appear to be at the foundation of inflammation seen in chronic diseases including aging, diabetes, renal disease, and cancer [33,34]. The ability to fully control a dog’s daily food intake and document dietary AGE intake in a laboratory setting may allow additional insights into human AGE research, as this level of nutritional control is not possible in standard human nutritional trials. Additional studies are indicated to more fully understand the relationship between dietary AGE intake, inflammation, and impact on chronic disease development and prevention.

### 4.5. Histidine, Carnosine, Anserine, and Ergothioneine

Histidine is an essential amino acid that plays a structural role for proteins and a gluconeogenic role and is essential as a precursor of neuroactive and regulatory compounds such as histamine, anserine, and carnosine [35]. Dogs fed the treatment food had increased levels of histidine, which is expected since the treatment food contained higher levels of histidine vs. the control food (3.27 vs. 1.82 g per 1000 kcal). Since the dogs were fed higher levels of histidine in the treatment group, it is not surprising to see increased levels of carnosine and anserine since both are histidine-containing di-peptides and synthesized endogenously. The higher levels of carnosine and anserine observed in the treatment group could also be the result of the dogs consuming chicken and chicken liver, since plant-based ingredients do not contain either metabolite [36,37,38,39,40,41]. Carnosine and anserine play numerous roles in the body. Carnosine and anserine, along with other metabolites of proteogenic amino acids (i.e., taurine and creatine) and omega 3 fatty acids (i.e., DHA), play important roles in brain development, cognitive health, behavior, and mood of dogs [41]. Both metabolites have also been shown to play a role in pH buffering in muscle for faster recovery, serving as antioxidants against lipid peroxidation and for enzyme regulation [36,42]. It is important to note that dogs consuming the higher levels of histidine in the treatment group did not have any differences from the control group for 1-methyl-histidine, a metabolite of histamine metabolism, indicating that histamine metabolism was not different between the groups.

A noteworthy metabolite that results from histidine metabolism is ergothioneine. Ergothioneine is an antioxidant currently being researched for oxidative stress, disease prevention, ocular health, neurological disorders, skin protection, and as a longevity nutrient in humans [43,44]. The authors are unaware of studies evaluating the pharmacokinetics of ergothioneine in dogs. Currently, fungi, blue-green algae, some bacteria, and yeast are only known to produce ergothioneine; therefore, it must be obtained from the diet regardless of species. Chicken liver has been reported to be as high as 140 ppm vs. 1–3 ppm in chicken breast and likely results from the chicken’s diet [45]. Dogs consuming the treatment food had increased levels of ergothioneine when compared to the control food, which could be explained by the chicken liver in the treatment diet.

The study had a few limitations, including the sample population consisting of a small number of geriatric dogs representing a single breed that were not controlled for comorbidities (primarily musculoskeletal pain issues) between groups and being performed in a controlled environment. The control food and fresh food diets had differing essential nutrient and macronutrient profiles, which makes some direct comparisons of the metabolic changes directly attributable to the processing method rather than the food format more challenging; however, processing issues related to AGE formation and exposure were quite evident.

## 5. Conclusions

Senior dogs fed fresh, human-grade food demonstrated increased metabolites consistent with ketosis, including BCAA metabolism, increased fatty acid β-oxidation, and lipolysis. Serum EPA and DHA were increased in the fresh food treatment group, which can be explained by the inclusion of chia seeds and salmon oil in the treatment food. This supports the use of ingredients high in ALA as metabolic precursors to EPA and DHA in dogs. The fresh food treatment group had decreased concentrations of serum AGEs compared to the control group. Senior dogs demonstrated dramatic changes in metabolism after 30 days when transitioned from a kibble diet to a fresh food diet, which were sustained during the year-long feeding period, showing healthy adaptation to a lower carbohydrate, minimally processed food. Implications regarding improved antioxidant status (carnosine, anserine, and ergothioneine), healthy ketosis (BHBA and BCAA ketoacids), and reduced serum AGEs on longevity and disease prevention should be investigated further as it relates to generalized inflammation and immune status using similar dietary approaches.

## Figures and Tables

**Table 1:** Nutritional profiles of the control and treatment foods.

		Control ^1^	Treatment ^2^
Nutrient Composition, per 1000 kcal			
Crude Protein	g	82.4	120.3
Alanine	g	6.17	6.63
Arginine	g	4.26	6.55
Aspartic acid	g	6.47	9.8
Cystine	g	1.22	0.72
Glutamic acid	g	12.98	16.25
Glycine	g	5.96	6.82
Histidine	g	1.82	3.27
Isoleucine	g	3.28	4.03
Leucine	g	7.99	7.57
Lysine	g	5.96	9.24
Methionine	g	1.98	1.75
Phenylalanine	g	3.80	3.96
Proline	g	6.05	4.68
Serine	g	3.62	4.59
Threonine	g	2.98	4.52
Tryptophan	g	0.64	1.59
Tyrosine	g	2.80	2.87
Valine	g	3.77	4.51
Taurine	g	0.15	0.31
Crude Fat	g	53.2	66.4
Linoleic Acid 18:2	g	12.4	11.9
Alpha-Linolenic Acid 18:3	g	0.6	5.9
Eicosapentaenoic Acid + Docosahexaenoic Acid	g	0.0	0.6
Minerals			
Calcium	g	4.56	4.30
Chloride	g	3.47	2.39
Copper	mg	39.51	4.22
Iodine	mg	5.17	0.82
Iron	mg	1155	46
Magnesium	g	0.33	0.40
Manganese	mg	176.3	5.1
Phosphorus	g	2.61	3.35
Potassium	g	1.70	3.51
Selenium	mg	1.37	0.21
Sodium	g	1.55	0.96
Zinc	mg	578	44
Vitamins			
Vitamin A	IU	79,027	15,694
Vitamin D	IU	12158	210
Vitamin E (Alpha Tocopherol)	IU	623	49
Thiamine (B1)	mg	42.6	6.2
Riboflavin (B2)	mg	45.6	9.2
Niacin (B3)	mg	334.3	55.8
Pantothenic Acid (B5)	mg	76.0	24.5
Pyridoxine (B6)	mg	51.67	2.64
Folic Acid (B9)	mg	10.03	0.51
Vitamin B12	mg	0.27	0.18
Choline	mg	5319	1028
Crude Fiber	g	8.2	9.8
NFE ^3^	g	88	4.1
Energy Content			
Metabolizable Energy, kcal/kg		3290	1300
% protein, ME ^4^		27.3	35.0
% fat, ME		36.2	62.0
% carbohydrate, ME		36.5	3.0

^1^ Control food (Lab Diet^®^ High Density Canine Diet 5L18) ingredient list: Ground Corn, Chicken Meal, Corn Gluten Meal, Rice Flour, Porcine Meat and Bone Meal, Dried Plain Beet Pulp, Poultry Fat Preserved with Mixed Tocopherols, Porcine Animal Fat Preserved with BHA and Citric Acid, Brewers Dried Yeast, Hydrolyzed Poultry By-Products Aggregate, Spray Dried Animal Blood Cells, Dried Egg Product, Dried Whey, Salt, Dicalcium Phosphate, L-Lysine, Calcium Carbonate, Soybean Oil, Natural Flavor, Potassium Chloride, Choline Chloride, Pyridoxine Hydrochloride, Menadione Dimethylpyrimidinol Bisulfite (Vitamin K), DL-Methionine, Cholecalciferol (Vitamin D3), Lecithin, Vitamin A Acetate, DL-Alpha Tocopheryl Acetate (Vitamin E), Ferrous Sulfate, Rosemary Extract, Inositol, Preserved with Mixed Tocopherols, Zinc Oxide, Calcium Pantothenate, Folic Acid, Thiamine Mononitrate, Calcium Iodate, Ethoxyquin (a Preservative), Riboflavin Supplement, Nicotinic Acid, Manganous Oxide, Vitamin B12 Supplement, Copper Sulfate, Cobalt Carbonate, Biotin. ^2^ Treatment food (The Farmer’s Dog Chicken Recipe) ingredient list: Chicken, Chicken Liver, Broccoli, Cauliflower, Brussels Sprouts, Chia Seeds, Tricalcium Phosphate, Salmon Oil, Potassium Chloride, Salt, Choline Bitartrate, Taurine, Magnesium Amino Acid Chelate, Zinc Amino Acid Chelate, Iron Amino Acid Chelate, Vitamin E Supplement, Potassium Iodide, Selenium Yeast, Vitamin B12 Supplement, Copper Amino Acid Chelate, Manganese Amino Acid Chelate, Riboflavin Supplement (Vitamin B2), Thiamine Mononitrate (Vitamin B1), Vitamin D3 Supplement, Pyridoxine Hydrochloride (Vitamin B6), Folic Acid. ^3^ Nitrogen Free Extract (Carbohydrate) = 100% − % moisture − % crude protein − % crude fat − % crude fiber − % ash. ^4^ Metabolizable energy (ME) basis is the percentage of total calories in the diet provided from protein, fat, or carbohydrate.

**Table 2:** Dietary impacts on serum amino acids and intermediates of amino acid metabolism.

Biochemical Name	Treatment Control				
	Month 0	Month 1	Month 3	Month 6	Month 12
creatine	1.38	**4.70**	**6.27**	**5.46**	**5.74**
creatinine	**0.86**	**0.79**	**0.89**	**0.87**	**0.87**
taurine	1.07	0.99	**1.32**	1.10	**1.25**
urea	0.92	1.30	1.24	1.23	**1.40**
leucine	1.01	**1.13**	**1.19**	**1.14**	**1.25**
4-methyl-2-oxopentanoate	**0.83**	**1.55**	**1.60**	**1.58**	**1.33**
isoleucine	1.08	**1.69**	**1.81**	**1.71**	**1.71**
3-methyl-2-oxovalerate	0.88	**3.42**	**2.97**	**3.38**	**2.54**
2-hydroxy-3-methylvalerate	1.23	**0.94**	**1.52**	**1.02**	**2.45**
valine	1.00	**1.85**	**1.81**	**1.69**	**1.83**
3-methyl-2-oxobutyrate	0.83	**2.76**	**1.88**	**2.81**	**1.94**
alpha-hydroxyisovalerate	0.80	**1.96**	**1.74**	**1.53**	**2.15**

Increase vs. control—red = *p* < 0.05 and pink = 0.05 < *p* < 0.10. Decrease vs. control—green = *p* < 0.05 and light green = 0.05 < *p* < 0.10.

**Table 3:** Dietary impacts on fatty acid beta-oxidation and fatty acid synthesis.

Sub Pathway	Biochemical Name	Treatment Control				
		Month 0	Month 1	Month 3	Month 6	Month 12
Ketone Bodies	3-hydroxybutyrate (BHBA)	1.23	**2.72**	**2.22**	**2.89**	**2.46**
Glycerolipid Metabolism	glycerol	0.74	1.31	**1.21**	1.24	1.27
	glycerol 3-phosphate	1.03	1.16	**1.24**	**1.42**	**1.38**
Fatty Acid Synthesis	malonate	0.87	**0.71**	**0.70**	**0.74**	**0.63**
Saturated Fatty Acid	butyrate/isobutyrate (4:0)	1.07	**1.69**	**2.08**	**1.74**	**1.60**
	myristate (14:0)	0.93	1.19	1.48	**2.78**	1.38
	palmitate (16:0)	1.15	1.01	**1.47**	**2.09**	1.15
	stearate (18:0)	1.36	0.91	1.15	**2.00**	1.06
	arachidate (20:0)	1.59	0.95	1.18	**2.21**	1.24
Acylcarnitines	acetylcarnitine (C2)	0.87	1.17	1.06	**1.32**	**1.26**
	hexanoylcarnitine (C6)	0.79	**1.51**	**1.53**	**1.66**	1.29
	palmitoylcarnitine (C16)	1.06	**1.43**	**1.28**	**1.69**	**1.35**
	stearoylcarnitine (C18)	1.25	**1.54**	**1.25**	**1.63**	**1.40**
	arachidoylcarnitine (C20)	**1.39**	**1.57**	**1.41**	**1.75**	1.27
	lignoceroylcarnitine (C24)	1.26	**1.60**	**1.72**	**1.98**	**1.42**
	cerotoylcarnitine (C26)	1.20	**1.38**	**1.69**	**1.65**	**1.42**

Increase vs. control—red = *p* < 0.05 and pink = 0.05 < *p* < 0.10. Decrease vs. control—green = *p* < 0.05.

**Table 4:** Dietary impacts on long-chain fatty acid metabolism.

Biochemical Name	Treatment Control				
	Month 0	Month 1	Month 3	Month 6	Month 12
linoleate (18:2n6)	1.10	1.14	1.23	**1.84**	0.89
linolenate alpha or gamma (18:3n3 or 6)	1.12	**5.87**	**5.58**	**21.26**	**9.11**
arachidonate (20:4n6)	1.43	0.68	1.23	**2.11**	0.95
hexadecatrienoate (16:3n3)	0.55	**5.93**	**4.99**	**9.91**	**6.29**
stearidonate (18:4n3)	0.62	**4.17**	**5.32**	**13.40**	**6.92**
eicosapentaenoate (EPA; 20:5n3)	1.61	**1.94**	**3.30**	**13.10**	**5.15**
heneicosapentaenoate (21:5n3)	1.23	**2.50**	1.57	**8.45**	**5.58**
docosapentaenoate (n3 DPA; 22:5n3)	2.09	1.17	1.76	**4.60**	1.83
docosahexaenoate (DHA; 22:6n3)	1.69	**1.44**	**2.41**	**7.83**	**3.70**

Increase vs. control—red = *p* < 0.05 and pink = 0.05 < *p* < 0.10.

**Table 5:** Dietary impacts on sugars and advanced glycation end products.

Sub Pathway	Biochemical Name	Treatment Control				
		Month 0	Month 1	Month 3	Month 6	Month 12
Sugars	1,5-anhydroglucitol (1,5-AG)	0.67	**0.37**	**0.34**	**0.28**	**0.27**
	glucose	1.01	0.93	1.02	1.03	0.93
	sucrose	0.46	**0.29**	**0.12**	**0.33**	**0.37**
	mannose	1.13	**2.14**	**2.83**	**2.73**	**1.87**
Advanced Glycation End Products	N6-carboxymethyllysine	0.93	**0.32**	**0.37**	**0.39**	**0.54**
	pyrraline	0.95	**0.06**	**0.09**	**0.05**	**0.06**

Increase vs. control—red = *p* < 0.05. Decrease vs. control—green = *p* < 0.05.

**Table 6:** Histidine, carnosine, anserine, and ergothioneine.

Biochemical Name	Treatment Control				
	Month 0	Month 1	Month 3	Month 6	Month 12
beta-alanine	1.07	1.12	**1.25**	**1.18**	**1.23**
histidine	0.99	1.04	**1.17**	1.02	**1.13**
carnosine	0.92	**1.71**	**1.90**	**1.91**	**1.86**
anserine	**0.62**	1.27	**1.55**	**1.65**	**1.75**
ergothioneine	0.90	**2.25**	0.94	**2.19**	**1.79**

Increase vs. control—red = *p* < 0.05 and pink = 0.05 < *p* < 0.10. Decrease vs. control—green = *p* < 0.05.

## Data Availability

Restrictions apply to the availability of these data that support the conclusions of this article. Data were obtained from Metabolon, Inc., and are available from the authors with the permission of Metabolon, Inc.

## Abbreviations

The following abbreviations are used in this manuscript:

ME	Metabolizable energy
CBC	Complete blood count
PCA	Principal component analysis
BCAA	Branched-chain amino acid
BCAT	Branched-chain aminotransferase
BCKD	Branched-chain α-keto acid dehydrogenase
TCA	Tricarboxylic acid
TAG	Triacylglycerol
DAG	Diacylglycerol
MAG	Monoacylglycerol
SCFA	Short-chain fatty acid
BHBA	3-hydroxybutyrate
PUFA	Polyunsaturated fatty acid
EPA	Eicosapentaenoic acid
DHA	Docosahexaenoic acid
ALA	Alpha-linolenic acid
AGE	Advanced glycation end product
1-5-AG	1,5-anhydroglucitol
MR	Maillard reaction
CML	Nɛ-(carboxymethyl)-lysine
